# An Odd Coupling: Nietzsche and W.E.B. Du Bois on 21st Century Philosophy of Education

**DOI:** 10.1007/s11217-021-09799-0

**Published:** 2021-10-05

**Authors:** Charles C. Verharen

**Affiliations:** 1 Department of Philosophy, Howard University, 2400 6th Street NW, Washington, DC 20059 USA; 2 Washington, USA

**Keywords:** Nietzsche’s Elitist Education, Du Bois’ Democratized Education, Technology and Universal University Education, An African Model for 21st Century Philosophy of Education

## Abstract

This essay contrasts Nietzsche’s remarks on elite education with W.E.B. Du Bois’ demand for democratized education. The essay takes their remarks as springboards for a twenty-first century philosophy of education rather than an historical account of their philosophies. Both thinkers cultivated Kant and Hegel’s dream that the spirit of freedom guided by reason would unite all the world’s peoples. Both held that education was key to realizing the dream. Their judgments about qualifying for education separated them. Nietzsche insisted that only the elite should receive the fullest measure of education. Du Bois believed that in the future virtually every human being would receive a university-level education. The essay’s principal point is to show how contemporary technology can make Du Bois’ dream a reality. An African philosopher’s working model demonstrates a path to universal university education.

## Introduction

Coupling Nietzsche and W.E.B. Du Bois appears to be absurd at first glance. Nietzsche divided humanity into two classes: the masters and the herd. In his later years, Du Bois advocated the abolition of classes. Nevertheless, in his earliest unpublished writing, Nietzsche said that “philosophy seems to begin with an absurd notion…” (*Philosophy in the Tragic Age of the Greeks,* 1962/1873, Chapter II, 38). What joins both thinkers together is their vision of a glorious unification of humanity. Nietzsche’s *Wanderer and His Shadow* presents a metaphor of the entire human community becoming “a tree that overshadows the whole earth” while the earth nourishes this tree (*Human, All Too Human*, hereafter HATH 1996/1879 [189], 235). Du Bois envisioned a humanity united by a freedom grounded in universal advanced education (Du Bois 1973, 1990; Lewis 1993, 2009).

Only two decades separate Nietzsche and W.E.B. Du Bois. Nietzsche finished his last research in 1888 (Young 2011; Safranski 2003; Prideaux 2018). Du Bois completed nearly all the requirements for a PhD in economics at the University of Berlin in 1893 (Lewis 1993). Both thinkers cultivated Kant and Hegel’s dream that the spirit of freedom guided by reason would unite all the world’s peoples (Kant 1903/1795; Hegel 1956/1837; see Bohman and Lutz-Bachmann 1997). Both held that education was key to realizing the dream. Their judgments about qualifying for education separated them. Nietzsche held that only the elite should receive the fullest measure of education. Du Bois believed that in the future virtually every human being would receive a university-level education.

The essay proposes a consilience of Nietzsche’s sense of philosophy’s aim and method with Du Bois’ insistence on universal university-level education. An African philosopher, Godfrey Nzamujo, has developed a working model in impoverished rural Africa that demonstrates how Nietzsche and Du Bois’ philosophies of education may be reconciled. The essay’s hypothesis is that philosophy’s mission is to guarantee life’s future on earth by destroying old values and replacing them with new ones that have more promise to execute philosophy’s mission. Nietzsche states this hypothesis in occasional remarks, but the essay does not claim that it represents his historical philosophy of education.

Nietzsche’s remarks on the philosophy of education are critical for addressing the existential crises created by contemporary globalization: catastrophic climate change, the sixth mass extinction, weapons of mass destruction, global terrorism and pandemics (Jamieson 2014; Gardiner 2011; Kolbert 2014; Schell 2000). In the preface to his early lectures on education, Nietzsche insists that philosophy must be a “meditation” on possible types of futures for humanity (*On the Future of Our Educational Institutions*, 2016/1872, hereafter FE, 94; Reitter et al. 2015). In the *Genealogy of Morality* Nietzsche claims that philosophers should serve as “guarantors of the future” (*Genealogy of Morality,* hereafter GM, 2014/1887, III [14], 91; see Stegmaier 2016). Rather than searching for universal truth, the *Gay Science* claims that philosophy must issue commands that promote “health, growth, future, power, life” (*Gay Science,* hereafter GS, 2001/1887, 2nd Edition Preface [2], 6; see Clark 1990). The *Gay Science* also establishes Nietzsche’s sense that the ultimate foundation for morality is “what benefits the preservation of the human race. Not from a feeling of love for the race, but simply because within them nothing is older, stronger, more inexorable and invincible than this instinct—because this instinct constitutes *the essence* of our species and herd” (Nietzsche’s emphasis, GS I [1], 27; see Ridley 2016, Leiter 2000).

Nietzsche claims that philosophy’s synoptic vision gives him the power to see far into the future. Affirming Plato’s (*Republic* 534b) definition of philosophy as synoptic vision, the philosopher is “a lookout at the top of the mountain, posted between today and tomorrow.” Philosophers are the “firstlings and premature births of the next century, to whom the shadows that must soon envelop Europe really should have become apparent by now” (GS Book V [343]: 199).

In his own mind Nietzsche is the first philosopher to realize that humanity’s increasing scientific and technological prowess may lead to self-extinction after twentieth century wars of unimaginable scope. Years before the Wright brothers’ first flight, he predicted that aircraft would be instruments of those wars (*Human All Too Human* 1996/1879). Philosophy’s task though its “mountain top” vision is to guarantee life’s future by discarding old values and creating more powerful new ones. Philosophy’s history narrates old values’ demolition and new values’ emergence from the foundations of the old (Rosenow 1989). Nietzsche envisions these new philosophical values as “experiments” on the path to transform philosophy into science, a centuries-long process that would “eclipse all the great projects and sacrifices of history to date. So far science has not yet built its cyclops-buildings; but the time for that will come too” (GS I [7], 34–35). In the past, experimental failure created cultural extinction. In twenty-first century existential crises, experimental failure means life’s extinction. Against that catastrophe, Nietzsche promotes a philosophical education that can train students to create solutions to unsolved problems (Bingham 2001; Church 2006). Nietzsche held that only the most elite students could profit from such an education.

The early W.E.B. Du Bois concurred with Nietzsche. He was determined to secure an elite education for himself and he believed that only those with his gifts, the “talented tenth,” merited such an education. As a youth he wanted to attend Harvard, the most prestigious university in the U.S. in his time. He was advised to attend Fisk University, an historically Black college in Tennessee as being more suited to his station in life. After receiving his BA from Fisk, he received a second BA from Harvard, majoring in philosophy under the tutelage of William James. After graduate work in history at Harvard, he completed nearly all requirements for his PhD in economic history at the University of Berlin, then the most prestigious university in the world. Initially impressed by Hegel, his experience of systemic racism turned him toward Marx and a socialist idea of universal equality (Du Bois 1920, 1940, 1968; Lewis 1993, 2009).

This essay contrasts the remarks of Nietzsche and Du Bois not as evidence of their historical philosophies of education but as springboards for a twenty-first century philosophy of education competent to address our contemporary existential crises. Their remarks serve as “intuition pumps” in Daniel Dennett’s sense (2014). If Nietzsche’s claim stated above that “philosophy seems to begin with an absurd notion” is correct, then imagination must be coupled with reason to enable philosophy to carry out its mission. Following Nietzsche’s insistence that his followers should reject his philosophy by reason of changing circumstances, the essay takes Nietzsche remarks as stimuli or “intuition pumps” for reconceiving philosophy’s task in an age of existential crises. The essay’s first section describes Nietzsche’s vision of all the world’s peoples united in a single body politic guided by a united European enlightened protectorate armed with a philosophical education. The second section addresses Du Bois’ arguments on behalf of universal university education. The third section shows how contemporary technology can help realize Du Bois’ “impossible” dream. The final section presents Godfrey Nzamujo’s working African model that demonstrates how that dream can become real.^1^

## Nietzsche’s Remarks on Education for the Elite

A provocation for the essay is Jonas and Yacek’s contention in *Nietzsche’s Philosophy of Education: Rethinking Ethics, Equality and the Good Life in a Democratic Age* (Chapter 5, The Doctrine of the Order of Rank, 99–119) that Nietzsche’s philosophy of education, stripped of its nineteenth century classism, provides a platform for a democratized education (see Blake et al. 2012; Cooper 2010). Nietzsche’s numerous remarks state to the contrary that a philosopher’s education is a privilege granted only to a favored few. Whether such remarks represent Nietzsche’s fundamental convictions is not a question that this essay addresses. The essay emphasizes Nietzsche’s anti-democratic remarks in order to construct an educational model that opposes them.

Nietzsche stated that the force of an education for the privileged elite would enable a unified European culture to take command of the whole earth. His prophetic powers are extraordinary. The globalism that binds the earth together a century after Nietzsche’s death emerges from European education’s focus on the science and technology that now threaten life on earth.

Can that same science and technology provide a platform for a democratized education? In FE’s Introduction*,* Nietzsche contends that grounds must have already been prepared in order for change to take place: No one should prognosticate about the future of culture and education “if he cannot prove that this culture of the future is to some extent already present and need only assert itself much more strongly….” (FE, 91). Jonas and Yacek argue that the grounds for democratizing education can be found in what they call Nietzsche’s “qualified egalitarianism” (2018, 102). However, Nietzsche’s remarks oppose democratic education from his earliest to latest writings. Technology’s power to make a philosophical education universally available was not yet evident in the nineteenth century. Nietzsche declared that the “press, the machine, the railway, the telegraph are premises whose thousand-year conclusion no one has yet dared to draw” (“Wanderer and His Shadow,” HATH [278], 378). Nevertheless, his remarks did not envision the human potential revealed in twentieth century liberation movements that challenged nineteenth century sexism, racism and classism.

Nietzsche’s formative thinking directed itself to ancient Greek culture. The classism of that culture would not permit universal schooling at any level. Nietzsche’s biographer, Rüdiger Safranski, states that Nietzsche sanctioned child labor, “noting with approval that Basel permitted children over the age of twelve to work up to eleven hours a day.” He also spoke against adult education (2003, 148–149). Nietzsche took both his philosophical method and his elitism from ancient Greece. Scholars have interpreted his sexist, racist and classist remarks that echo Greek sentiments as hyperbolic or ironic (see Jonas and Yacek, endnotes 1–10, 117–119 for an extensive list). This essay takes those remarks at face value in order to construct an educational model that opposes them. In numerous explicit remarks, Nietzsche with Aristotle advocates androcentrism, elevating men over women (Aristotle 1941). He goes even further to promote ethnocentrism, imagining that the collective European nations through their Greek heritage will lead the world to a thousands of years European *Reich.* Like Plato and Aristotle and many of his own nineteenth century contemporary Europeans, Nietzsche states that humans can be divided into masters and slaves by reason of their diverse capacities for leadership (Plato 1966).

Nietzsche remarked that an enslaved humanity would willingly follow their European masters. The 2nd edition of the *Gay Science* (GS Part V [377], 241) advocates a “new slavery—for every strengthening and enhancement of the human type also involves a new kind of enslavement—doesn’t it?” And unlike Aristotle who imagined that slavery could be replaced by machine technology, Nietzsche stated that slavery was an inborn trait. While Nietzsche multiplied sexist remarks only after his traumatic relationship with Lou Salomé (he nearly committed suicide), his remarks are resolutely racist and classist from the 1871 “Greek State” to the 1888 *Ecce Homo* Nietzsche (1871/1872).

Rejecting the “*Deutschland über Alles”* of his own times that was to become Nazi Germany’s catchword, Nietzsche proposed a united *Europa über Alles.* As early as *Human, All Too Human,* he announces that “man has to set himself ecumenical goals embracing the whole earth” (HATH [25], 25). He delegates this task to the “good” Europeans whose “great task” is the “direction and supervision of the total culture of the earth” (WS [87], 332). In the late notebooks he foresaw emergent international dynasties dedicating themselves to “the task of rearing a master race, the future ‘masters of the earth.’” This “aristocracy” will “last for thousands of years” by reason of the “will of philosophical men” who will use “democratic Europe” as the mechanism controlling “the destinies of the earth.” Such aristocrats possess such extraordinary power because of the “superiority of their willing, knowing, wealth and influence.” Nietzsche predicts a time “when we will learn to think differently about politics” (*Writings from the Late Notebooks* 2003/1885–1888, 2 [57], 71).

Critics may suggest that Nietzsche reserved this kind of intemperance for his private thoughts. Nevertheless, in *Ecce Homo* he castigates German nationalism for provoking wars that temporarily “cheated” Europe out of a “*force majeure…*strong enough to make Europe into a unity, a political *and economic* unity for the purpose of world governance…” (EH “The Case of Wagner,” [2] 140). Taking a page from Charles Darwin’s cousin, Francis Galton, Nietzsche in the same text advocates for a eugenics capable of “breeding humanity to higher levels (which includes the ruthless extermination of everything degenerate and parasitical).” Nietzsche promises a “tragic age” brought about by the “harshest though most necessary wars” (EH “The Birth of Tragedy” [4], 110). Against the claim that such effusions result from Nietzsche’s terminal dementia in the fall of 1888, Aaron Ridley, editor of the Cambridge *Ecce Homo,* claims that the text’s hyperbole dramatizes an accurate expression of Nietzsche’s conclusive thoughts (2005/1888, Introduction, vii–xxxiv).

Saturated in the sexism, racism and classism of nineteenth century Europe, Nietzsche’s published remarks do not present a way forward that would destroy the barrier between master and herd. Liberation movements challenging those three “isms” in the intervening centuries should remind us that Nietzsche begs us to repudiate his own work in order to create better avenues to the future. *Human, All Too Human* speaks of the danger that a “great thinker” who believes he possesses “absolute truth” will become “a tyrant” (HATH [261], 124). Calling himself a herald rather than a knight, Nietzsche advises his readers to create their own philosophies: “At least be readers of this book, so that later, through your actions, you can consign it to destruction and obliteration” (FE [Preface], 95). The problem is that the three “isms” have been characteristic features of humans at least since the dawn of recorded history and perhaps longer. What hope is there of overcoming them? Is the glorious unification of humanity even remotely possible?

History discloses humanity’s proclivity to increase the size of its groups. Thinkers like the Buddha and Christ pleaded for humanity to form itself into a single unified group. Kant, Hegel and Marx saw human unification as an inevitable progression. Darwin (1936/1871) argued that expansion of group definition emerges from evolutionary processes. He made two claims about conditions for survival (see Johnson 2010; Richardson 2004; Ridley 2016). First, groups that increase their numbers have a competitive edge. Second, groups that bind their members tightly together increase their chances for survival. Humanity moves in the direction of increasing group size, from familial units to tribes to tribal nations to nation states to empires. Nietzsche joined Kant, Hegel and Marx (the latter unknowingly!) in thinking that a glorious future could unite all humanity into a single group. So, there is evidence of a historical movement that could buttress their collective optimism. More importantly, contemporary threats of mass extinction of life present humanity with a choice we have never had before. We can unite as a single group to guarantee life’s future or we can exterminate ourselves. One ironic reason for optimism is the current Covid-19 pandemic. The world must unite to counter this threat and guard against subsequent zoonotic viruses that may be even more virulent.

Combatting the Covid-19 pandemic requires virtually all humans to engage in a struggle against a mortal enemy. However, the pandemic will run its course, whether stopped by effective vaccines or by killing millions before herd immunity is established. Challenging the other existential crises will require creating new knowledge on a scale the world has not yet imagined. There is no clear path to stopping catastrophic climate change. The Harvard biologist E. O. Wilson (2016) recommends returning half of the earth’s land surface to nature in order to halt the sixth mass extinction of life, but current political and economic paths to that goal are absent. Nations continue to develop nuclear, chemical and biological weapons of mass destruction. No current practice prevents nations, non-governmental groups or individuals from deploying these weapons in acts of terrorism. For the first time in *Homo sapiens*’ 300,000 life span, humans have acquired the ability to create the sixth mass extinction. Nature created the first five mass extinctions. Only a creative humanity bonded together as one group may have the power to stop the sixth.

Against Malthusian fears about the overpopulation of the earth, Nietzsche’s *Wanderer and His Shadow* presents a metaphor of the entire human community becoming “a tree that overshadows the whole earth” while the earth nourishes this tree. To achieve this goal, “whole nations, whole centuries toil to discover and *thoroughly to test* new methods for promoting a great human collective….” The task requires much suffering. In the best case, select individuals through their “wisdom and prudence” will create “the measures adopted by whole nations and whole ages.” The path is perilous as humanity possesses no “infallible instinct” for choosing methods that prevent its destruction. Nevertheless, we must “*look in the face* our great task of *preparing* the earth for the production of the greatest and most joyful fruitfulness – a task for reason on behalf of reason!” (Nietzsche’s emphasis, HATH [189], 356).

## Du Bois’ advocacy for universal university education

Proposing a radical philosophy of higher education, W.E.B. Du Bois (1973) imagined a time when virtually all humans would have a university education: “Today there is but one rivalry between culture and vocation, college training and trade and professional education and that is the rivalry of Time. Someday every human being will have college training” (*The Education of Black People* 1973, 106).

Du Bois advocated an education for all humans that would unleash their creative potential. He argued that African American colleges and universities have a unique ethical obligation to serve as the spearhead to universal higher education: They “can show the majority the way of life.” He wrote in 1941 that “salvation and culture” come not from “overwhelming rich and powerful groups” but from the “still small voice of the oppressed and the determined who knew more than to die and plan more than mere survival” (1973, 177).

In Nietzsche’s time, one that overlapped with that of the young Du Bois, Du Bois’ prophesy was preposterous. In our time, it is imperative. Humanity’s crises are so dire that virtually every human must be working toward solutions to those crises. Du Bois notes that in his time universities’ elitism separated humans into two classes: the educated and the uneducated: “This is the problem of education with which the world is most familiar, and it tends to two ends: it makes the mass of men dissatisfied with life and it makes the university a system of culture for the cultured” (1973, 85). He foresaw a future in which all humans would be able to exercise the university’s primary function: research to solve humanity’s unsolved problems (Verharen et al. 2014b). In his philosophy of democratic education, “the marvelous talent and diversity and emotion of all mankind” will flourish to the degree that the “university reaches down to the mass of universal men and makes the life of normal men the object of its training…” (1973, 86–7).

Du Bois began to overcome his earlier restriction of university education to the “talented tenth” on his first trip to Africa: “Once upon a time some four thousand miles east of this place, I saw the functioning of a perfect system of education. It was in West Africa, beside a broad river…” (1973, 83). He observed this “perfect system,” developed in “Yorubas and other Sudanese and Bantu” villages where “education was completely integrated with life. There could not be uneducated people. There could be no education that was not at once for use in earning a living and for use in living a life” (1973, 84).

Du Bois gradually overcame his opposition to Booker T. Washington’s philosophy of industrial training for African Americans in historically Black colleges in the southern United States. Anticipating the African working model for a “united college and vocational school” described in the essay’s next section, Du Bois’ reformed university would graduate alumni “who can think clearly and function normally as physical beings; who have a knowledge of what human life on earth has been, and what it is now; and a knowledge of the constitution of the known universe” (1973, 76).

The curriculum for such a university mirrors the curriculum that Nietzsche proposed for training students to achieve the synoptic vision characteristic of philosophers. The curriculum includes all seven intellectual disciplines building upon the pure abstractions of mathematics, logic and grammar (the traditional 3 R’s of grammar school) and extending to the global reach of the fine and technical arts, history, science and philosophy itself. Against Nietzsche’s conception of the ancient Greek origin of this curriculum, Du Bois traces its general outlines back to Africa in earliest recorded history: “The riddle of existence is the college curriculum that was laid before the Pharaohs, that was taught in the groves by Plato, that formed the trivium and quadrivium, and is to-day laid before the freedmen’s sons by Atlanta University” (1990, 64).

Du Bois believed that African Americans have a mission to make “the ideal of human brotherhood…a practical possibility, a mission “to civilization and humanity, which no other race can make” (1995, 26.) His argument is based on the idea that those who suffer most from a problem have the strongest incentive to solve the problem. He believes the “black votes of Reconstruction” were instrumental in developing public school systems in the southern United States. The impetus came from Black Union troops in Missouri “fighting the last battle of the West” who conceived of systematic education of their fellows in their home state. … The public school system of the whole South is the gift of black folk” (1973), 129).

In Du Bois’ philosophy of education, a primary mission of historically Black colleges and universities (HBCUs) is to serve as the spearhead for universal university education (Verharen et al. 2020). In the last century a number of HBCUs developed continuing education programs to reach African Americans who had not been able to continue their education past high school. Anna Julia Cooper, the first African American to receive a PhD in philosophy from the Sorbonne, dedicated her house near Howard University in Washington, DC to adult education. Alain Locke, the first African American to receive a Rhodes scholarship and the first to receive a PhD in philosophy from Harvard, championed the cause of adult education in his research. Carter G. Woodson wrote extensively on how adult education might offset the “miseducation of the Negro” (Locke 1989; Grant et al. 2016). Howard discontinued its continuing education programs toward the end of the last century because of funding problems. More generally, underfunding and impoverished endowments prevent the more than 100 HBCUs in the United States from taking the first steps to execute Du Bois’ dream of universal university education through adult education.

Morehouse College, an HBCU in Atlanta, Georgia, has inaugurated a program to help students who have not finished their Morehouse degrees while in residence to take online courses in order to graduate: “As an online student, you will be immersed in a community designed to make your voice heard and gain a life-changing academic experience right on your laptop or mobile device” (Morehouse College 2021). Morehouse establishes a model that other HBCUs may follow to help realize Du Bois’ ambition for HBCUs spearheading the drive to universal university education. However, a much bolder model is required, one that capitalizes to the very fullest on technology’s potential to achieve the dream.

## Nietzsche and Du Bois: An Odd Coupling

Are the dreams of Nietzsche and Du Bois purely philosophical imaginings, far removed from any chance of becoming real? Ever the realist, Nietzsche believed that humanity as the universe’s creation has only a brief time on the stage. Our “loftiest task” for the time we have left is one “growing together into oneness and commonality, so that mankind can confront its impending doom as a united entity….” This single goal “encompasses the sum total of all the ennoblement of the human being” (*Untimely Meditations* Part IV, Richard Wagner in Bayreuth [4], translated by Safranski 2003, 105).

While pairing these two philosophers may seem obtuse, a common thread joins them: the unification of a flourishing humanity. Their approaches to this goal are quite different. Du Bois wrote that through universal higher education’s power, classism, racism and sexism will vanish. Nietzsche stated that a united Europe will unite humanity, while preserving the distinctions of class, race and sex. Given the conjunction of advanced technology and twentieth century liberation movements, we can discard Nietzsche’s elitism. What we cannot dismiss is his sense of philosophy’s aim and method. Philosophers capitalize on synoptic vision to synthesize a picture of the whole of human experience. Armed with this “love of wisdom,” philosophers acquire the competence to destroy decadent values and replace them with new creations that have the promise to guarantee life’s future.

Nietzsche sensed his original incapacity for this task because of his ignorance of the sciences. He committed himself to the principle that philosophy as synoptic vision must be grounded in science—to say nothing of the other humanities as well as religion and mythology. In the *Nachlass* he asks whether a philosopher in his times is “still *possible*.” Given the explosion of the sciences, it is most likely that the philosopher “will never achieve an *overview*” (Nietzsche’s emphasis, *Unpublished Fragments Spring 1885-Spring 1886,* 35 [24], 88).

Impressed by Nietzsche’s focus on synoptic vision, the American pragmatist Richard Rorty, one of Nietzsche’s keenest disciples, claims that philosophers as individuals are no longer capable of executing philosophy’s mission. The breadth and depth of knowledge required for commanding a secure path to life’s future requires expertise in a wide range of disciplines. Teamwork binding those disciplines together in pursuit of life’s future must be philosophy’s own future. Resisting contemporary efforts to replace philosophy with STEM disciplines, Rorty insists that “philosophy always buries its undertakers” by reason of its synoptic vision (*Philosophy as Poetry* 2016, 61). Robert Frodeman and Adam Briggle’s *Socrates Tenured: The Institutions of 21st Century Philosophy* (2016) join Rorty in proposing a model of “field philosophy” to join philosophy with other disciplines in order to address unsolved problems with a focus on social justice.

## Technology’s Role in Democratizing Philosophical Education

Nietzsche insists that we destroy his philosophy and build a better one out of his tumbled-down foundations stones. We cannot destroy his sense of philosophy as a “mountain top” or synoptic view because philosophy has served that role since its inception. Given his prescience about human irrationality’s potential to destroy the species, we also cannot reject his claim that philosophy’s primary mission is to guarantee life’s future. However, we now have the instruments to reject his nineteenth century conviction, spurred in part by the research of Malthus, Darwin and Spencer, that nature divides humanity into classes superior and inferior to one another (see Desmond and Moore 2009; Kendi 2019).

Three new technologies now make it possible to dismiss Nietzsche’s remarks favoring classism: green energy, machine labor and information communication. Nietzsche realized that he could not anticipate what wonders technology would bring in the future. The *Wanderer and His Shadow* announces the twenty-first century hammer that may strike a death blow to nineteenth and twentieth century sexism, racism and classism: “*Premises of the machine age.* – The press, the machine, the railway, the telegraph are premises whose thousand-year conclusion no one has yet dared to draw” (HATH [278], 378). While Nietzsche predicted the catastrophic wars of the twentieth century, his imagination about the “premises of the machine age” did not foretell that age’s consequences for a philosophical education.

Competition for scarce resources, whether the scarcity is real or perceived, has created the conditions that promote classism. Contemporary technologies have the potential to reduce that competition. Robotics, AI and other machines have made it possible for ordinary citizens to enjoy the luxuries that medieval kings possessed. With the twelve-hour workday and child labor now formally abolished (although still widely practiced), Nietzsche’s “herd” now has access to the *sine qua non* of creative endeavor: leisure. “Free time,” the necessary condition for *school* in the Greek sense of leisure, is no longer as scarce for many in the world as it was in Nietzsche’s time. The work week has declined from 72 to 40 hours and even less in Western European countries. The “machine age” has diminished scarcity to the point that a United States presidential candidate promoted universal base income (UBI) as a human right—a quantum leap for schooling as leisure if humanity survives our existential challenges.

United States politicians now advocate a “Green New Deal” that can reduce energy scarcity. Solar energy can not only reduce CO_2_ and methane emissions, but work toward the infinite recycling of other resources (McDonough and Braungart 2002). Abundant, inexpensive energy can be used to recycle formerly scarce material “from cradle to cradle.”. The “machine age” has the potential to reduce the amount of labor that does not require creative human consciousness. Most importantly, information communication technology with the expansion of broadband access has the potential to offer universal education to virtually all the world’s peoples. With existing appropriate technologies, superabundance has the promise of replacing scarcity.

Nietzsche claims that in times of superabundance, humans begin to create in unimaginable ways. “Variation,” Darwin’s evolutionary engine, “[s]uddenly…arrives on the scene in the greatest fullness and splendor…and the individual dares to be individual and to stand out.” That same “‘individual’ now stands there, compelled to a legislation of his own, to his own arts and wiles of self-preservation, self-enhancement, self-redemption” (*Beyond Good and Evil* 2014/1886 [262], 176–177, cited in Jonas and Yacek, 147).

## An African Working Model for a Democratized Philosophical Education

Optimists like the Harvard psychologist Stephen Pinker make the claim that advances in technology promote humanity’s ethical progress (Pinker 2011, 2013, 2018). While his research is controversial, the dreams of Nietzsche and Du Bois about the glorious unification of humanity cannot be realized without extraordinary advances in technology. As a kind of “mountain-top” or synoptic vision, philosophy must incorporate technology into its visions of a future that has the promise of guaranteeing life’s future. Critically important to this concept of philosophy that embraces virtually all the intellectual disciplines in its method is the inclusion of thinkers that have not been included in the company of professional philosophers. The prominent feminist philosopher Virginia Held believes that philosophy has begun to attack “real problems…such as global poverty, or terrorism and political violence or environmental issues” (2018, 154). She reasons that “women and others previously missing in philosophy” encourage philosophy to address “new topics previously ignored” (2018, 142). Du Bois is an African American philosopher advancing that agenda with his sense of HBCUs’ mission for advancing education for those most in need.

An African philosopher, Godfrey Nzamujo, also stands as an example of those “others previously missing in philosophy.” His path to creating a working model that can realize Nietzsche and Du Bois’ dreams of humanity’s unification rests on the foundation of contemporary advanced technology. His ecovillage model accords with Du Bois’ conviction that traditional African villages provide examples of a “perfect education” where “[t]here could not be uneducated people” (1973, 83–84).With Du Bois, Nzamujo holds that contemporary “[u]niversity culture today is largely elitist” (2018, 7). African universities in particular have not realized their full potential to solve their communities’ problems. One possibility is that those universities be restructured “to serve society by spurring efforts to generate knowledge, innovations, ideas and cultures commensurate with the scale, scope and complexity of the challenges that confront Africa today” (Nzamujo 2018, 1). However, Nzamujo believes that a better solution is to embed the university in the African villages that most desperately require a university-level education to solve their existential problems. That solution rests upon Nzamujo’s deployment of technology.

Nzamujo, a Dominican priest from Nigeria, exercises the multiplex roles of the field philosophy method in his own person. Trained in mathematics, philosophy, theology, microbiology, computer engineering and international development, Nzamujo joined these disciplines together to create a model for solving rural poverty in Africa. After graduate work at the University of California/Irvine and teaching at Marymount College in Los Angeles, Nzamujo secured 44 hectares of land on the edges of Porto Novo, Benin.

Working on the field philosophy model, he established a village grounded in principles of agroecology (Songhaï Center 2018). Green solar and biomass technologies move the village to a carbon neutral footprint. Capitalizing on the maxim that “waste is wealth,” the center practices “cradle to cradle” recycling (McDonough and Braungart 2002). A USAID study notes that “Songhaï integrates 'zero waste' and ‘total productivity’ concepts” (Vodouhe and Zoundji 2013, 3). The government of Benin and the United Nations have provided financial support for the Centers (United Nations Development Program 2008). The Economic Community of West African States recognizes Songhaï as a regional center of excellence for Africa (de Luca et al. 2013; Songhaï.org 2020).

Nzamujo conceives of the village as a “knowledge enterprise.” A primary capital cost for setting up the village was investment in information communication technology. Broadband furnishes access to the global store of knowledge. The internet replaces the libraries and textbooks missing from the world’s most impoverished areas (see Bowen 2013). From its inception in 1988 in Benin, the model has spread to more than fifty other sites in seventeen African countries. The villages are called *Songhaï Centers* to commemorate the West African Songhaï Empire (Nzamujo 2002).

Nzamujo blends Nietzsche and Du Bois’ philosophies of education in several ways. Like Du Bois, he believes that higher education is a universal human right. His expertise in communication technology allows him to work toward realizing Du Bois’ dream in the world’s most impoverished continent. Like Nietzsche, he believes that a philosophical education is the only instrument that can guarantee life’s future—both for the individual and for humanity itself (Verharen et al. 2014a, 2021). Against Nietzsche and with Du Bois, he holds that virtually every human being has the capacity to accomplish the primary task of a university—research. For Nzamujo, there can be no conflict between the university’s research and teaching missions. Students serve as apprentices mastering the profession of research in the diverse fields that justify calling an institution a *university*.

Nzamujo’s closest affinity with Du Bois springs from his development of the Songhaï Leadership Academy. The Academy is open both to Songhaï village residents and others who wish to learn how to replicate the Songhaï model. Its purpose, in Nzamujo’s words, is “to serve society by spurring efforts to generate knowledge, innovations, ideas and cultures commensurate with the scale, scope and complexity of the challenges that confront Africa today” (2017, 1). Based on the method of field philosophy, the Academy follows Nietzsche’s principle that an education for philosophers must be grounded in broad areas of experience. Responsible for raising their own food, the Songhaï Center members are close to nature as the engine of their lives.

The Academy offers technology courses covering all aspects of organic agriculture together with limited production of agricultural machinery and techniques for the distribution and export of food products (Nzamujo, Songhaï Leadership Academy Bulletin, 2017). The theoretical courses cover the basic principles of the STEM fields, as well as the humanities. Weaving together the sciences, technologies and humanities through synoptic philosophical vision, the curriculum “challenges us and engages us to learn from the basic principles of the workings of our planet for more than three billion years (Nzamujo 2018, 20). The curriculum features biomimicry and eco-design technologies to foster sustainability. His conviction is that “many of our technological problems have already been solved in nature in elegant, efficient and ecologically sustainable ways’ (Nzamujo 2018, 10).

The Songhaï Centers serve as seedbeds for their own replication, as their growth into seventeen African countries in the past thirty-two years demonstrates. Nzamujo is also using information communication technology to distribute the knowledge created in the Songhaï Leadership Academy to broader African populations. Using USAID funding, Songhaï started “a network of community teleservice operations starting in 1999” together with a radio communication system linking the centers “to give the population in general and farmers in particular access to new information technologies” (Vodouhe and Zoundji 2013, 2).

Nzamujo’s Songhaï Center demonstrates the possibility of democratizing a philosophical education in the world’s poorest regions. It at once promises the hope of realizing Du Bois’ dream of universalized university education and Nietzsche’s dream of guaranteeing life’s future. Relying on solar and agricultural technologies for energy, the Songhaï model fashions a global path to arrest catastrophic climate change. Guided by its maxim that ‘waste is wealth’ and fueled by green energy technologies, a globalized Songhaï model would diminish the 300,000-year-old human competition for scarce resources. By teaching the creation of new knowledge from primary through post-secondary education, the Songhaï model offers what the better angels of our nature most want: in Nietzsche’s sardonic words, “the earthly happiness of all” (*The* *Birth of Tragedy and Other Writings* 1999/1885, [18], 86, cited in Safranski 2003, 146).

## Conclusion

A philosophy of education for the twenty-first century will highlight several of Nietzsche’s remarks about philosophy’s aim and method. A review of twentieth century liberation movements will force the reexamination of Nietzsche nineteenth century conception of the populations qualified to exercise philosophy’s powers. Unlike his philosophical predecessors, Nietzsche predicted that human irrationality could derail humanity’s progress toward freedom. Neither Kant nor Hegel imagined that science and technology unchecked by philosophy could lead to humanity’s self-extinction. Primed by Darwin’s account of the extinction of species, Nietzsche remarked that the primary task of philosophy is to guarantee life’s future. Philosophy’s method is to destroy and replace old values that no longer serve that mission. Even in his earliest unpublished writing, he saw unchecked science as the instrument of self-extinction: “The goal of science is the destruction of the world” (Notebook P 1 15, *Nietzsches Werke* IX, 72, cited in *Philosophy and Truth,* 1979/1872–1876, hereafter PAT, fn. 9, 156). As stated above, Nietzsche continued that theme in the *Gay Science,* and made it the primary focus of part III of the *Genealogy of Morals.*

With another boost from philosophy’s synoptic vision, Nietzsche conjured up a united Europe that would use its collective power to lead the world to a unified paradise. Kant, Hegel and Marx projected that glorious vision, but did not specify Europe as its executor. Hegel, for example, declared that “America is therefore the land of the future, where, in the ages that lie before us, the burden of the World’s History shall reveal itself—perhaps in a contest between North and South America.” The “tired old Europe” that bored Napoleon did not have the power to bring the world to true freedom (*The Philosophy of History* 1956/1837, 86). The globalization that now conquers the earth is the heritage of the European-inspired development of science and technology. Nietzsche was correct in predicting a European conquest of the earth but his optimism about the result was misplaced. Unchecked science and technology resulted not in the *freedom* envisioned by that German “Gang of Four,” but the *freedoom*, if a neologism is permitted, of the existential crises threatening life.

Oddly enough, one of those crises, the global pandemic, shows the way to save life. Until virtually every human being has joined the struggle against Covid-19, vast numbers of lives remain at risk. Epidemiologists predict that Covid-19 is simply a precursor to much more virulent strains. The urgency for a global response to the virus may hasten the global community’s awareness that universal action is required to address the other existential threats: catastrophic climate change, the sixth mass extinction, weapons of mass destruction, and terrorism, whether governmental or group, on a global scale.

The long-range key to dissolving those threats lies in Du Bois’ dream of universal university education. The irony is that only science and technology can make that dream real. The difficulty lies in figuring out how to make philosophy into the tool that can check science—Nietzsche’s vision of philosophy’s mission. In his earliest unpublished writing as stated above, Nietzsche said that “the goal of science is the destruction of the world” (PAT, fn. 9, p. 156). Only philosophy has the power to stop that destruction: “It is not a question of annihilating science, but of *controlling* it.” Nietzsche claims that science is totally dependent on philosophy “for all its goals and methods, though it easily forgets this.” Philosophy’s method is to set the values that control science: “…*philosophy which gains control also has to consider the problem of the level to which science should be permitted to develop: it has to determine value”* (Nietzsche’s emphasis, PAT Sect. 28, p. 8). A philosophy of education for the twenty-first century demands that philosophy reclaim its role of controlling science.

## Footnotes

1 Portions of this essay have appeared in my previous publications, where they were embedded in different contexts and aimed at different audiences. This essay explores the consilience between Nietzsche and DuBois' philosophies of education and presents an African model for putting that consilience into practice. The previous articles focused on the genealogies of human values (2020a), the future of ethics and education (2020b), and African environmental ethics (2021) coauthored with my research team.
